# Ethik und Corona: Normative Grenzen politischer Maßnahmen zur Eindämmung der Covid-19-Pandemie

**DOI:** 10.1007/s41358-020-00240-5

**Published:** 2020-11-09

**Authors:** Dagmar Schulze Heuling

**Affiliations:** Staatswissenschaftliche Fakultät, Universität Erfurt, Erfurt, Deutschland

## Abstract

Die Maßnahmen gegen die Covid-19-Pandemie sind einschneidend. Begründet und gerechtfertigt werden sie mit einem ethischen Argument: dem Schutz des menschlichen Lebens. Doch während konkrete Maßnahmen Gegenstand intensiver Auseinandersetzungen sind, fehlen Betrachtungen des Diskurses aus der Metaperspektive. Dieser Artikel leistet dazu einen ersten Beitrag. Er zeigt auf, dass die vorherrschende Argumentationsstruktur die einer Güterabwägung ist. Dabei wird jedoch der Tatsache, dass die Maßnahmen zur Verhinderung der Infektionsausbreitung nicht nur schützen, sondern selbst Todesfälle verursachen, nicht genügend Rechnung getragen. Unter Rückgriff auf das Trolley-Dilemma sowie das Bundesverfassungsgerichtsurteil zum Luftsicherheitsgesetz wird gezeigt, dass die Corona-Pandemie die Politik vor die Wahl stellt, entweder das Sterben von Menschen nicht zu verhindern, oder aber andere Menschen zu diesem Zweck zu opfern. Letzteres ist ethisch bedenklich und verfassungswidrig. Daraus folgt, dass einige der verfügten Maßnahmen zur Eindämmung der Pandemie in Frage zu stellen sind. Daraus folgt jedoch nicht, dass jedes Handeln unmoralisch wäre. Daher schließt der Artikel mit Überlegungen zu Möglichkeiten und Grenzen einer politischen Reaktion auf die Pandemie.

## Abstract (en)

Around the globe, drastic measures have been implemented in order to contain the Covid-19 pandemic. Ultimately, their justification is an ethical one: the duty to save human lives. But while individual policies are intensely discussed, an analysis of the discourse from a meta-perspective remains a desideratum. This paper seeks to contribute to such an analysis. It shows that structurally, the discussion is engaged in a risk-benefit-analysis. Within this analysis the fact that the containment policy itself causes deaths is insufficiently taken into account. In paralleling the current situation with the trolley problem and a ruling of the German Constitutional Court concerning the Luftsicherheitsgesetz (Aviation Security Act), the paper argues that the pandemic presents politicians with the choice to either not prevent the death of some, or sacrifice the lives of others in order to save them. As the latter is ethically dubious and unconstitutional, some of the policies already implemented need to be reevaluated. However, the argument does not imply that any action to mitigate the effects of the Covid-19 pandemic is illegitimate. Thus, the paper closes by examining the ethical conditions for political action in this case.

## Einleitung

Die Maßnahmen zur Eindämmung der Corona-Pandemie haben das Leben in Deutschland in zuvor ungekanntem Maß verändert. In allen Lebensbereichen wurden massive Einschränkungen verfügt, darüber hinaus sind die Menschen aufgerufen, ihre Wohnungen möglichst nicht zu verlassen und auf persönliche Kontakte zu verzichten. Diese tiefgreifenden Eingriffe und ihre noch kaum absehbaren Folgen werden mit einem ethischen Argument gerechtfertigt: dem Schutz von Menschenleben.

Der ethische Diskurs muss folglich eine erhebliche Begründungslast schultern. Dementsprechend breit und intensiv werden die Debatten über das Für, das Wider und das richtige Maß der Einschränkungen geführt. Es wird abgewogen, welchen Erfolg Maßnahmen versprechen, welche Kosten damit einhergehen, was zumutbar und zu rechtfertigen ist. Diese vorherrschende Logik der Abwägung ist angesichts der Problemstellung naheliegend.

Allerdings, so argumentiere ich, ist diese Argumentationsstruktur im vorliegenden Fall nicht zulässig. Unter Rückgriff auf die Figur des Trolley-Dilemmas zeige ich, dass die Abwägungslogik im Kern darauf hinausläuft, das bewusste Opfern von Menschenleben zu rechtfertigen. Das ist sowohl ethisch als auch verfassungsrechtlich bedenklich.

Im nachfolgenden Text gebe ich zunächst einen sehr kurzen Überblick über die bisherige Entwicklung sowie die aktuelle Situation (2.). Auch wenn die Fakten hinlänglich bekannt sein dürften, scheint dies im Angesicht der sich rapide ändernden Daten- und Erkenntnislage zur Situierung der Argumentation (Stand Ende April 2020) unerlässlich. Im nächsten Schritt zeige ich, dass die Beiträge in der gegenwärtigen Debatte über geeignete und vertretbare Maßnahmen zur Bewältigung der Pandemie implizit oder explizit die Logik der Güterabwägung bemühen, wenngleich mit unterschiedlichen Ergebnissen und Akzentuierungen (3.1). Eine Analyse der Argumentationsstruktur macht deutlich, dass dem die Auffassung zu Grunde liegt, dass die ethische Herausforderung der Situation darin besteht, kollidierenden Pflichten gerecht zu werden (3.2). Das ist jedoch m. E. kein adäquates Verständnis der Situation. Ich argumentiere, dass diese Auffassung die negativen Folgen der Maßnahmen zur Infektionseindämmung möglicherweise übersieht, in jedem Fall aber nicht richtig einordnet (3.3).

Warum, entgegen der verbreiteten Ansicht, eine Abwägung im vorliegenden Fall ethisch unzulässig ist, verdeutliche ich anhand des Trolley-Dilemmas (4.) Nach einem kurzen Überblick über das Trolley-Dilemma, seine Varianten und Implikationen (4.1) lege ich dar, weshalb die Figur des Trolley-Dilemmas eine treffende Abstraktion der gegenwärtigen Bemühungen zur Eindämmung der Corona-Pandemie ist. In diesem Zusammenhang verweise ich zudem auf eine andere Aktualisierung des Trolley-Problems in der Form des Luftsicherheitsgesetzes (4.2).

Schließlich diskutiere ich, was die veränderte theoretische Perspektive für die praktische Politik bedeutet (5.). Der Text schließt mit einer kurzen Zusammenfassung.

## Die Situation

Spätestens seit Dezember 2019 war in einer chinesischen Provinz eine ungewöhnliche Häufung schwerer Lungenentzündungen festgestellt worden. Diese wurden durch ein bisher nicht bekanntes Virus aus der Familie der Coronaviren, SARS-CoV‑2, ausgelöst. Auf Grund des Erregers sowie des erstmaligen Auftretens wird die Erkrankung als Coronavirus Disease 2019, kurz Covid-19, bezeichnet. Nachdem die Krankheit in den ersten Monaten des Jahres 2020 in immer mehr Ländern auftrat, stufte die World Health Organization (WHO) den Ausbruch von Covid-19 am 12. März 2020 als Pandemie ein.

Inzwischen vermelden Behörden, Universitäten und andere Forschungsinstitute in kurzen Abständen immer neue Infektionszahlen und Todesopfer. Trotz intensiver Forschungstätigkeit ist das Wissen über SARS-CoV‑2 noch sehr gering. Einschätzungen über Infektionswege, geeignete Behandlungsmaßnahmen sowie die Gefährlichkeit des Virus liegen weit auseinander und ändern sich innerhalb kurzer Zeit.

Zur Illustration: Die Johns Hopkins University, die eine umfangreiche Datenbank zu Covid-19 mit weltweitem Fokus betreibt, geht für Deutschland von einer Letalität des Virus von 3,4 % aus.^1^ Bei einer ungehinderten Ausbreitung könnte das für Deutschland Millionen von Todesopfern bedeuten. Eine ähnliche Einschätzung spricht aus den Äußerungen von Regierungsmitgliedern.^2^ Die Bundeskanzlerin bringt es auf den nüchternen Nenner: „Es ist ernst.“ (Merkel 2020) Für andere Länder werden sogar noch deutlich erschreckendere Zahlen als für Deutschland angegeben. So beziffert die Johns Hopkins University die Case-Fatality-Ratio für Belgien mit knapp 15 %.^3^

Zu einer deutlich anderen Einschätzung gelangt Anthony Fauci, der das National Institute for Allergy and Infectious Diseases der USA (NIAID) leitet. Er geht davon aus, dass tödliche Verläufe in etwa 0,1–1 % der Fälle zu erwarten sind (Fauci et al. 2020).^4^ In absoluten Zahlen befürchtet er für die USA zwischen 100.000 und 200.000 Tote. Übertragen auf die Bevölkerungsgröße Deutschlands würde dies etwa 25.000–50.000 Todesopfern entsprechen.^5^

Am unteren Ende der kursierenden Schätzungen bewegt sich auch das vorläufige Ergebnis der ersten Bevölkerungsstudie von SARS-CoV‑2 Infektionen und Covid-19-Erkrankungen in Deutschland. Für die Gemeinde Gangelt, einen der sogenannten Hotspots des Infektionsgeschehens in Deutschland, wurde eine Letalität von 0,37 % ermittelt (Streeck et al. 2020).

Diese Zahlen zeigen, dass die Corona-Pandemie selbst im besten Fall als eine ernste Bedrohung der Gesundheit gelten muss. Bisherige Erkrankungsmuster zeigen darüber hinaus, dass bestimmte Gruppen besonders gefährdet sind. Während Kinder nahezu nicht und junge Menschen vergleichsweise selten bzw. leicht erkranken, treten bei Menschen mit Vorerkrankungen, zumal wenn sie im höheren Lebensalter sind, schwere und tödliche Krankheitsverläufe sehr viel häufiger auf. Zugleich dürfen die Zahlen und die täglich neu gewonnenen Erkenntnisse nicht darüber hinwegtäuschen, dass das Wissen über SARS-CoV‑2 immer noch sehr gering ist. Weder ist geklärt, warum ein kindlicher Organismus offenbar ganz anders reagiert als ein erwachsener, noch, wie hoch die Zahl der Infizierten ist – und damit bleibt die Gefährlichkeit des Virus Objekt von Spekulationen. Auch ein Impfstoff oder Gegenmittel sind noch nicht bekannt.

Inzwischen haben viele Länder zu drastischen Maßnahmen gegriffen, um die Ausbreitung der Infektionen und damit der Krankheit einzudämmen (Cheng et al. 2020). Dazu zählen Ausgangs- und Kontaktverbote, Verbote von Veranstaltungen aller Art, das Schließen von Schulen und Kindergärten, Geschäften, Restaurants und Bars sowie von Museen, Bibliotheken, Sportstätten und dergleichen mehr.

## Die Diskussion über die Antipandemiepolitik

Ziel dieses Kapitels ist es, die Diskussion über die Antipandemiepolitik darzustellen, die darin verwandten Argumentationsmuster herauszuarbeiten und deren sachliche, vor allem aber ethische Implikationen zu analysieren. Das konkrete Vorgehen folgt der Logik eines ethischen Urteils. Ähnlich einem allgemeinen syllogistischen Schluss beruhen praktisch-ethische Urteile auf zwei Komponenten: einem ethischen Prinzip und der Berücksichtigung aller relevanten Fakten (Tännsjö 2013, S. 4). Eine Analyse des ethischen Diskurses über die Antipandemiepolitik muss daher, um dieser Doppelstruktur gerecht zu werden, sowohl die relevanten Fakten der realen Situation als auch die ethischen Prinzipien, wie sie in der Argumentation zum Ausdruck kommen, betrachten.

### Positionen in der Debatte

In Deutschland wurden die Einschränkungen des öffentlichen Lebens, Eingriffe ins Wirtschaftsleben, Kontaktverbote und andere Beschneidungen von Freiheitsrechten innerhalb kürzester Zeit verfügt. Insofern konnte diesen Maßnahmen keine breite Debatte vorausgehen, eine solche entwickelte und entwickelt sich vielmehr weitgehend parallel zu ihrem Gegenstand. Wie angesichts von Umfang und Schwere der Maßnahmen nicht anders zu erwarten, wird der Kurs des weitgehenden Lockdowns sehr kontrovers diskutiert. Gleichwohl war und ist, vermutlich angesichts der gefühlten Größe der Bedrohung, in vielen Medien ein durchaus verständnisvoll-befürwortender Tenor auszumachen. Umfragen zufolge hält auch ein großer Teil der Bevölkerung in Deutschland diese Politik für richtig, wenngleich die Zustimmung im Zeitverlauf zurückgeht. (COVID-19 Tracker 2020; Blom et al. 2020).

Kritische und vermittelnde Stimmen, die die Verhältnismäßigkeit der Maßnahmen nicht ohne weiteres als gegeben ansehen, nehmen allerdings zu. Einige fragen grundsätzlich nach der Zulässigkeit, zumindest aber der Verhältnismäßigkeit so tiefgehender Freiheitseingriffe (Lüpke 2020). Andere weisen auf die negativen Begleiterscheinungen hin, etwa die wirtschaftlichen Folgen des Lockdowns. Immer zahlreicher werden die Hinweise auf Gruppen, die in besonderer Form unter der Suspendierung des bisherigen Lebens leiden. Dazu zählen u. a. psychisch Kranke (Stalinski 2020), von häuslicher Gewalt Betroffene (Neurologen und Psychiater im Netz 2020), Kinder aus Familien, die ausfallenden Schulunterricht nicht kompensieren können (Hurrelmann und Dohmen 2020) oder Sterbende und ihre Lieben, denen die Möglichkeit des Abschiednehmens genommen wird (Fleischhauer 2020).

Betrachtet man abstrakt, also unabhängig vom konkreten Argument, die Struktur der Beiträge, fällt auf, dass die Argumentation in aller Regel der Logik der Abwägung folgt. Abwägen bedeutet, die Vor- und Nachteile möglicher Optionen genau zu bedenken, oder die Ergebnisse alternativer Handlungsweisen sorgfältig miteinander zu vergleichen. In der Grundrechtsdogmatik ist die Abwägung für die Frage nach der Verfassungswidrigkeit von Grundrechtseingriffen von zentraler Bedeutung. Dabei muss die Wertigkeit des Zwecks der Schwere des Eingriffs und dem Grad der Zielerreichung gegenübergestellt werden. Letztendlich gilt es, verschiedene Handlungsoptionen miteinander zu vergleichen und die Variante mit dem geringsten Schaden zu wählen. Schaden ist dabei als weit gefasster Begriff zu verstehen, der gesundheitliche, ökonomische, soziale, kulturelle und rechtliche Aspekte, mithin negative Folgen aller Art, beinhaltet. Eine Abwägung bedeutet also mitnichten, dass der Wert eines Lebens in Geld ausgedrückt werden soll. Was allerdings bei einer Abwägung geleistet werden muss, und darin besteht die Herausforderung, ist, Schäden ganz unterschiedlicher Natur kommensurabel zu machen.

Explizit lässt sich dies der ad-hoc Stellungnahme des Deutschen Ethikrats entnehmen (Deutscher Ethikrat 2020). Laut dem Ethikrat besteht der ethische Kernkonflikt zwischen dem Ziel, die Funktionsfähigkeit des Gesundheitssystems zu sichern, und den schwerwiegenden Nebenfolgen der Maßnahmen, die der Zielerreichung dienen sollen (Deutscher Ethikrat 2020, S. 2). Seine Lösung „erfordert eine gerechte Abwägung konkurrierender moralischer Güter“ (Deutscher Ethikrat 2020, S. 2 vgl. auch S. 5). Hier wird die Abwägung also explizit als Lösungsstrategie des bestehenden Konflikts identifiziert.

In einem Kommentar fordert Anna Klöpper in der taz vom 22. März 2020, also parallel zum Inkrafttreten der bisher höchsten Stufe der Einschränkungen: „Es ist jetzt Zeit, dass die Frage nach der Verhältnismäßigkeit der Maßnahmen mit ein wenig mehr Nachdruck gestellt wird, als bisher.“ (Klöpper 2020) Der Hinweis auf die Verhältnismäßigkeit beinhaltet die Erkenntnis, dass Maßnahmen gegen die Corona-Pandemie nicht kosten- und folgenlos sind. Daher gilt es, verschiedene Handlungsoptionen gegeneinander abzuwägen. Unabhängig davon, was sich letztlich als beste Lösung darstellen wird, ist der Text eine Warnung, dass der vorhandene Konflikt zumindest nicht ohne Diskussion, ohne genaue Prüfung der Verhältnismäßigkeit, nach einer Seite hin aufgelöst werden darf. „Die Politik sollte sich jetzt sehr genau überlegen, ob sie die Daumenschrauben weiter anzieht und die Grundrechte etwa auf Bewegungs- und Versammlungsfreiheit noch massiver einschränken will.“ (Klöpper 2020).

Parallele Überlegungen stellt Jasper von Altenbockum in der Frankfurter Allgemeinen Zeitung an (Altenbockum 2020). Neben einer übermäßigen Einschränkung von Freiheitsrechten benennt der Kommentar den Konflikt zwischen Gesundheit und der Gefahr einer zerstörten Gesellschaft, und ganz allgemein das Problem, dass „die Linderung der Not auf der einen Seite eine neue Not auf der anderen Seite schaffen kann“ (Altenbockum 2020). Damit findet sich hier ebenso wie in Klöppers taz-Kommentar als unhinterfragte Grundlage der Argumentation die Abwägung. Allerdings kommt von Altenbockum zu einem anderen Ergebnis. Für ihn ist ein möglichst rigider Lockdown einschließlich allgemeiner Ausgangssperren richtig, denn der „Hammer“ ist nach seiner Ansicht der schnellste Weg durch die Pandemie und damit auch durch die Einschränkungen hindurch.

Im Ergebnis nicht unähnlich, aber mit einer anderen Akzentuierung der Beweggründe argumentiert der Ministerpräsident von Baden-Württemberg, Winfried Kretschmann. Er beruft sich darauf, dass es eine Wertehierarchie gäbe, in der Leben und Gesundheit an oberster Stelle stünden (SWR Aktuell 2020). Eine Abwägung muss also zugunsten solcher Maßnahmen ausfallen, die Leben und Gesundheit schützen, woraus sich Kretschmanns befürwortende Haltung für strikte Schutzmaßnahmen und seine Skepsis gegenüber Lockerungen erklären.

Kretschmanns Parteikollege, der Tübinger Oberbürgermeister Boris Palmer, mahnt hingegen öffentlich, die Folgen der gewählten Antipandemiemaßnahmen nicht zu übersehen. Der Preis für die Rettung von Menschen, die möglicherweise „in einem halben Jahr wegen ihres Alters oder wegen schwerer Vorerkrankungen sowieso tot wären“ sei den UN zufolge weltweit verschärfte Armut sowie der Tod von einer Millionen Kindern (Frühstücksfernsehen 2020, Abschn. 3:10–3:28). Diese Äußerung löste öffentliche Empörung aus. Palmer lehnte eine Entschuldigung zunächst ab, stellte aber später klar, dass er niemals das Lebensrecht älterer oder kranker Menschen in Frage stellen würde (Hüsgen 2020).

Sieht man von der Auseinandersetzung über Palmers Wortwahl ab, so zeigt sich auch hier wieder deutlich die Argumentationsfigur der Abwägung. Wenn mehr Menschen an den Nebenwirkungen eines Medikaments sterben als das Medikament Menschen retten kann, dann muss laut Palmer die Abwägungsfolge sein, das Medikament abzusetzen oder geringer zu dosieren – übersetzt in politisches Handeln bedeutet das, passgenauere Maßnahmen zu entwickeln, die ebenfalls Infektionen verhindern, aber weniger gravierende Nebenfolgen haben (Frühstücksfernsehen 2020).

Die umstrittene Äußerung Palmers fiel in einer Sendung, deren Thema ein Interview war, das Bundestagspräsident Wolfgang Schäuble dem Tagesspiegel gegeben hatte (Birnbaum und Ismar 2020). Schäuble spricht darin explizit von einem Abwägungsprozess zwischen Einschränkungen und Lockerungen. Die Auffassung, dass das menschliche Leben einen unantastbaren Höchstwert darstelle, sei so nicht richtig. Der Jurist argumentiert mit dem grundgesetzlichen System der Grundrechte, die sich gegenseitig beschränken. Das bedeutet nichts anderes, als dass Grundrechte sich relativ zueinander verhalten und folglich im Konfliktfall eine Abwägung stattfinden muss. „Wenn es überhaupt einen absoluten Wert in unserem Grundgesetz gibt, dann ist das die Würde des Menschen.“ (Birnbaum und Ismar 2020).

Auf die Menschenwürde als „unverrechenbaren“ Wert beruft sich auch ein Gastbeitrag in der Frankfurter Rundschau. Anders als der Titel suggerieren mag, ist die Berufung auf die Menschenwürde jedoch keine grundsätzliche Absage an jegliches Abwägen, sondern vielmehr ein Plädoyer für eine Differenzierung innerhalb der Gesamtbetrachtung einer Situation. „Abwägen kann man dabei entweder zwischen den wirtschaftlichen Kosten des einen und denen des anderen Bereichs; oder zwischen deren ethischer Zulässigkeit und Gebotenheit (Entsprechendes gilt auch für die anderen Bereiche); nicht aber zwischen Ethik und Wirtschaft.“ (Schmid Noerr 2020). Unabhängig von der Frage, ob sich diese Sphären in der Realität immer klar abgrenzen lassen, kommt hier auf normativer Ebene wiederum die Vorstellung einer Wertehierarchie zum Tragen, insofern im Ergebnis dem Schutz des Lebens eine alle anderen moralischen Güter überragende Position eingeräumt wird.

### Die Struktur der Debatte

Die Struktur der bisherigen Diskussion, das hat dieser kleine Querschnitt von Beiträgen gezeigt, geht davon aus, dass zwischen Bündeln von Gütern abgewogen werden muss. Während keine Zweifel an der Wertigkeit des Ziels bestehen – immerhin geht es um Gesundheit und Leben – gibt es unterschiedliche Einschätzungen der Gefahrenlage, der Schwere der Freiheitseingriffe sowie der wirtschaftlichen und sozialen Folgen. Aus der unterschiedlichen Gewichtung dieser Aspekte erklären sich unterschiedliche Meinungen.

Alle Beiträge gehen davon aus, dass unterschiedliche Güter bedroht sind, etwa Gesundheit, Versammlungsfreiheit, die Möglichkeit zu sozialer Interaktion und Nähe, Bildung und Erziehung von Kindern und Jugendlichen, Wohlstand oder gar die wirtschaftliche Existenz und andere mehr. Grundsätzlich sind alle diese Güter schützenswert. Daher korrespondiert mit ihnen eine moralische (und oft auch eine rechtliche) Pflicht, sie nicht zu verletzen. Die Herausforderung der gegenwärtigen Situation, das stellt etwa die Empfehlung des Ethikrates explizit klar, ist die Unmöglichkeit, allen diesen Pflichten zugleich gerecht zu werden. Es kommt mithin zu einer Pflichtenkollision (Deutscher Ethikrat 2020). Welches konkrete Maßnahmenbündel die geringstmögliche Pflichtverletzung nach sich zieht, wird höchst unterschiedlich beurteilt.

Einige Beiträge gehen davon aus, dass unterschiedliche Güter und damit unterschiedliche moralische Pflichten betroffen sind, es aber keine situationsunabhängige und objektive Hierarchie dieser Güter und Pflichten gibt. Diese mögen zwar tendenziell von höherem oder geringerem Wert sein – vor die Wahl gestellt, ob man einmal ihr Recht auf Leben oder ihr Briefgeheimnis verletzen solle, dürften die meisten Menschen eine eindeutige Präferenz haben. Sie sind aber immer auch situationsabhängig – vielleicht verzichtet eine Sterbende lieber auf einige Lebensminuten, als ein lebenslang gehütetes Geheimnis enthüllt zu sehen. Daher müssen immer alle Umstände betrachtet werden, einschließlich solcher Aspekte wie der erhofften Wirkung, der Dauer des Eingriffs, der Schwere des zu erwartenden Schadens oder dessen Reversibilität. Nach gebührlicher Berücksichtigung aller Faktoren ist gewissermaßen eine geistige Gesamtsumme zu bilden, die Aufschluss darüber gibt, welche Handlungsalternative die weniger schwerwiegende Verletzung moralischer Güter impliziert.

Andere Stimmen postulieren dagegen eine Güterhierarchie. Das führt dazu, dass Verletzungen von Leben und Gesundheit prinzipiell schwerer wiegen als Schäden anderer Art und somit einen größeren Einfluss auf das Abwägungsergebnis haben, das Ergebnis also in gewissem Maß präjudizieren.

Auf den ersten Blick könnte es scheinen, dass es sich bei dieser Variante der Argumentation gar nicht um eine Abwägung handelt.^6^ Die etwa vom baden-württembergischen Ministerpräsidenten Kretschmann explizit angerufene Hierarchie suggeriert vielmehr, dass es eine klare Rangfolge moralischer Pflichten gibt. Aufgabe der Politik wäre es dann lediglich, diese vorgegebene Wertentscheidung umzusetzen. Doch gegen diese Auffassung sprechen zwei Gründe. Erstens ist die angeführte Hierarchie eine subjektive Präferenz, und nicht die verbindliche Vorgabe einer moralischen oder rechtlichen Autorität. Zweitens würde eine solche Hierarchie, wenn es sie tatsächlich gäbe, eine konsequente Anwendung über alle Lebensbereiche hinweg verlangen. In Anbetracht der Gesundheitsrisiken, die von Alkohol- und Tabakkonsum ausgehen, müsste im ganzen Bundesland Rauch- und Trinkverbot herrschen, die Staatsweingüter Baden-Württembergs dürften allenfalls noch alkoholfreien Wein herstellen. Dass es keine ernsthaften Versuche gibt, solche Regelungen zu implementieren, zeigt, dass eine Güterhierarchie, die einer Abwägung nicht zugänglich ist und aus der sich daher schematisch das richtige Verhalten ableiten ließe, nicht existiert.

### Blinder Fleck

Die Unterschiede sollten nicht über die grundlegende Gemeinsamkeit nahezu aller Debattenbeiträge hinwegtäuschen. Durchweg wird eine Abwägung als notwendig empfunden. Will man dies nicht als unumwundenen Utilitarismus interpretieren (das würde m. E. der Intention der Beiträge widersprechen), dann impliziert die Verwendung dieses ethischen Prinzips, dass es eine Kollision moralischer Pflichten gibt, die nicht in einer eindeutigen Rangfolge stehen bzw. nicht absolut gelten. Es ist jedoch fraglich, ob die gegenwärtige Situation damit zureichend beschrieben ist. Ich argumentiere, dass die bisher in die Debatte einbezogenen Fakten durchweg einen Aspekt nicht angemessen würdigen. Sie stellen also keineswegs eine Bewertung aller relevanten Fakten dar, und können allein aus diesem Grund weder zu einem zutreffenden noch zu einem gültigen Urteil kommen.

Bei einer umfassenderen Betrachtung der Situation, einschließlich der unerwünschten Folgen der Corona-Verordnungen, drängt sich eine bittere Erkenntnis auf. Nicht nur Covid-19 kann Menschenleben fordern, auch die Verordnungen zur Eindämmung der Pandemie können tödliche Konsequenzen haben.^7^ Diese Realität klingt in vielen Beiträgen an. Dennoch wird sie bislang nicht explizit thematisiert, sondern ist ein blinder Fleck der Debatte.^8^ Ihr Ausblenden führt dazu, dass die ethischen Überlegungen am Kern des Problems vorbeigehen. Werden diese nicht intendierten Folgen der Antipandemiepolitik mit berücksichtigt, ergibt sich ein ganz anderes Bild. Die Situation stellt sich nunmehr als Dilemma dar, bei dem die Frage zu beantworten ist, ob es moralisch legitim oder sogar geboten ist, Menschenleben zu opfern, um andere Menschen zu retten. Dieses Dilemma tritt klar zutage, wenn man sich die negativen gesundheitlichen Folgen der Eindämmungsmaßnahmen vor Augen führt, die bis zum Tod reichen können. Die folgenden Beispiele wollen keine detaillierte Folgenabschätzung leisten. Vielmehr sollen sie die jeweiligen Mechanismen, ihre gesundheitsschädlichen und tödlichen Folgen verdeutlichen.

Der Aufruf, zu Hause zu bleiben, das Herunterfahren des öffentlichen Lebens und die Kontaktbeschränkungen führen zur deutlichen Reduktion von Bewegung im Alltag. Die Möglichkeit, dies durch Individualsport auszugleichen, ist nicht immer gegeben. Nicht nur Personen, die in häuslicher Quarantäne leben, leiden daher unter Bewegungsmangel. Als Mangel gilt bei Erwachsenen, wenn sie sich pro Woche weniger als zweieinhalb Stunden mit mittlerer Intensität bewegen. Kinder sollten sogar täglich mindestens eine Stunde toben, turnen oder laufen (WHO 2010, S. 7–8).

Bewegungsmangel gilt als einer der führenden Risikofaktoren für nicht ansteckende Krankheiten (Guthold et al. 2018). Ganz allgemein erhöht er das Risiko eines vorzeitigen Todes, insbesondere auf Grund von Herz-Kreislauf-Erkrankungen, Schlaganfall, Bluthochdruck, Brust- und Darmkrebs, Typ-2-Diabetes und Osteoporose (Warburton et al. 2010). Neuere Studien weisen überdies darauf hin, dass Bewegungsmangel für 3–8 % der Demenzfälle verantwortlich zeichnet (Sallis et al. 2016). Parallel muss man sich vor Augen führen, dass selbst Patientinnen und Patienten ohne Vorerkrankungen, die temporär mobilitätseingeschränkt sind (etwa durch die Verletzung einer Extremität und Angewiesensein auf Gehstützen), häufig präventiv mit Antikoagulantien behandelt werden, da die mit der Mobilitätseinschränkung einhergehende Verringerung der Muskelaktivität das Thromboserisiko erhöht (Encke et al. 2015). Somit lässt sich ermessen, dass diese Politik sowohl langfristig lebensverkürzend als auch unmittelbar lebensbedrohlich ist.

Auch die Eingriffe in die Wirtschaft, die bereits nach kurzer Zeit zu höherer Arbeitslosigkeit und Rezession geführt haben (Bundesagentur für Arbeit 2020), wirken sich auf die Gesundheit aus. So ist Arbeitslosigkeit mit einem schlechteren Gesundheitszustand und kürzerer Lebenserwartung assoziiert (Scholz und Schulz 2007), zwischen Rezession und Suizid besteht ein positiver Zusammenhang (Hawton und Haw 2013; Chang et al. 2013). Während diese Folgen weit überwiegend auf die ergriffenen politischen Maßnahmen zurückzuführen sind, stellt sich die weltweite Gesamtsituation komplexer dar. So ist die Zunahme der weltweiten Armut – die Weltbank geht in einer Schätzung davon aus, dass 40–60 Mio. Menschen in die extreme Armut abrutschen werden, womit ihre physische Existenz unmittelbar bedroht ist (Gerszon Mahler et al. 2020) – sowohl als Folge der Pandemie wie auch der ergriffenen Gegenmaßnahmen zu erklären.

Eine umfassende Folgenabschätzung hätte viele weitere Faktoren zu berücksichtigen. Dazu zählen etwa psychosoziale Folgen, wie Stress, Vereinsamung, Alkoholabhängigkeit oder häusliche Gewalt, ebenso wie die befürchtete Zunahme bzw. Verschlimmerung von psychischen Erkrankungen wie Depressionen, Angst- oder Zwangsstörungen. In welchem Umfang sich dauerhafte Entwicklungs- und Bildungsdefizite bei Kindern und Jugendlichen, insbesondere solchen mit spezifischem Förderbedarf, zeigen werden, ist eine noch offene Frage (Redaktionsnetzwerk Deutschland 2020).

Die Erkenntnis, dass die Maßnahmen zur Eindämmung der SARS-CoV-2-Infektionen selbst negative gesundheitliche Folgen haben, die bis zum Tod reichen können, verändert die Struktur der Situation und damit das ethische Problem, mit dem die Pandemie ganz zweifelsohne konfrontiert. Abstrakt beschrieben haben wir es mitnichten mit einer Gemengelage von Werten oder Gütern zu tun, aus der die unter den gegebenen Umständen bestmögliche Kombination zu wählen ist. Sondern es geht um die Frage, ob die Rettung von Menschenleben ein Handeln rechtfertigt, das andere Menschen, aber hoffentlich eine geringere Zahl, das Leben kostet.

In der bundesrepublikanischen Normenordnung ist diese Frage zu verneinen. Ein solches Vorgehen ist unvereinbar mit der Menschenwürde. Das hat das Bundesverfassungsgericht 2006 für eine analoge Situation, die Erlaubnis zum Abschießen von als Waffen missbrauchten Flugzeugen, klargestellt (1 BvR 357/05). In der ethischen Literatur werden dagegen unterschiedliche Auffassungen vertreten, und auch die moralische Intuition von Menschen kommt zu unterschiedlichen Antworten.

## Antipandemiepolitik als Trolley-Problem

Im vorigen Kapitel habe ich dargelegt, dass die Debatte über die Antipandemiepolitik implizit davon ausgeht, dass eine Abwägung zwischen konkurrierenden moralischen Gütern vorzunehmen ist. Diese Auffassung verdeutlicht einen blinden Fleck der Debatte: die nicht nur schweren, sondern zum Teil tödlichen Auswirkungen der Maßnahmen. Eine Berücksichtigung aller Handlungsfolgen erzwingt daher eine grundlegend andere Perspektive. Im Kern ist die gegenwärtige Situation ein moralisches Dilemma, bei dem sich die Frage stellt, ob man die Gesundheit (oder gar das Leben) einiger Menschen opfern darf, um die Gesundheit (oder gar das Leben) anderer, möglicherweise einer sehr viel größeren Zahl, zu schützen.

Antworten finden sich in der Auseinandersetzung mit dem Trolley-Dilemma beschrieben. Nachfolgend führe ich kurz theoretisch in das Trolley-Dilemma ein, ehe ich darlege, weshalb es sich bei der gegenwärtigen Situation um ein solches Dilemma handelt und welche ethischen Erkenntnisse sich daraus ergeben.

### Das Trolley-Dilemma – ein Überblick

Unter dem Begriff Trolley-Dilemma oder Trolley-Problem ist eine Gruppe hypothetischer Situationen bekannt, anhand derer die Implikationen unterschiedlicher ethischer Theorien und Entscheidungen verdeutlicht und analysiert werden können. Meist geht es um einen leeren, außer Kontrolle geratenen Straßenbahnwagen, den Trolley, der das Leben von Menschen bedroht.

In der wohl bekanntesten Variante ist der Trolley im Begriff, in eine Gruppe von fünf Personen zu rasen, die durch die Kollision sterben werden. Zwischen dem Trolley und den Menschen befindet sich allerdings noch eine Weiche. Ein Umstellen würde den Trolley auf ein anderes Gleis lenken, auf dem sich nur eine Person befindet. Ist es moralisch vertretbar oder sogar geboten, den Hebel der Weiche zu betätigen (Thomson 1985)?

Beschrieben wurde das Trolley-Dilemma zuerst von Philippa Foot (Foot 1978), einer Pionierin der angewandten Ethik. Foots Ursprungsgedanke unterscheidet sich allerdings von den später populär gewordenen Varianten. Bei ihr muss die Fahrerin der Straßenbahn sich entscheiden, ihr Fahrzeug entweder auf ein Gleis zu steuern, auf dem sich fünf Personen befinden, oder aber auf ein Gleis, auf dem nur eine Person zugegen ist.^9^

Zum Trolley-Problem ist sowohl theoretisch wie empirisch intensiv geforscht worden (vgl. Bruers und Braeckman 2014 für einen Überblick). Die empirische Forschung hat gezeigt, dass moralische Intuitionen nicht nur von ethischen Überzeugungen, sondern auch von kontingenten und moralisch nicht bedeutsamen Rahmenbedingungen beeinflusst werden, etwa dem Alkoholpegel oder der Frage, ob der Mensch, der den Trolley aufhalten wird, in einem Bus sitzt oder sich direkt auf den Schienen befindet (Duke und Bègue 2015; Waldmann und Dieterich 2007). Demgegenüber haben sich theoretische Arbeiten um eine konsistente Begründung der Entscheidung sowie der ethisch bedeutsamen Unterschiede zwischen den verschiedenen Varianten bemüht (Edmonds 2013).

Dabei stehen sich als idealtypische Pole deontologische sowie utilitaristische Ansätze gegenüber.^10^ Während eine deontologische oder Pflichtenethik das richtige Handeln danach beurteilt, ob allgemein gültige Regeln eingehalten wurden und also auf die Handlung selbst abstellt, kommt es für den Utilitarismus auf die Folgen der Handlung an, die möglichst gut sein sollen. Eine strenge Anwendung ergibt im Fall der utilitaristischen Ethik eine Präferenz für diejenige Handlungsweise, die den geringsten Schaden anrichtet, also im Trolley-Fall die wenigsten Todesopfer fordert. Das müsste dann allerdings auch in einer etwas abgewandelten Version des Trolley-Problems gelten. Darin kann der Trolley nicht durch das Umstellen einer Weiche gestoppt werden, sondern nur dadurch, dass ein sehr schwergewichtiger Mann von einer Brücke so auf die darunterliegenden Schienen gestoßen wird, dass sein Körper den Trolley aufhält (Thomson 1976, S. 207 f). Wie im Ausgangsbeispiel rettet der Tod eines Mannes fünf Menschenleben – aus utilitaristischer Perspektive ist es also vorteilhaft, den Mann auf die Schienen zu stoßen.

Eine deontologische Ethik kommt hingegen zum gegenteiligen Ergebnis. Weil eine Pflicht besteht, Menschen nicht zu töten,^11^ ist es aus dieser Perspektive ausgeschlossen, dass Menschenleben geopfert werden – auch dann, wenn andere und sogar mehr Menschenleben dadurch gerettet werden können. Anders, als ein flüchtiger Blick suggerieren könnte, besteht hier keine Pflichtenkollision. Denn die Bedrohung durch den Trolley ist eine fremdgesetzte Gefährdung. Wenn der Trolley die fünf Menschen auf dem Gleis überrollt, handelt es sich um einen Unfall. Grundsätzlich besteht zwar auch hier die Pflicht zur Hilfeleistung. In der konkreten Situation ist diese für die Weichenstellerin aber unmöglich, genauso, wie es beispielsweise bei einer verklemmten Weiche der Fall wäre. Dass die Unmöglichkeit hier auf der ethischen Norm des Tötungsverbots beruht, und nicht auf einem technischen Defekt, ändert nichts am Ergebnis. Das Nichtverstellen der Weiche ist ein Nichtrettenkönnen. Das Verstellen der Weiche ist ein Töten (so inzwischen auch Thomson 2008).

In den wenigsten Fällen folgt die moralische Intuition von Menschen konsequent einer der beiden Theorien. So halten es rund 90 % der Befragten für richtig, die Weiche zu verstellen, sodass der Zug auf das Gleis mit nur einer Person geleitet wird. Genauso viele halten es aber für falsch, den schwergewichtigen Mann von der Brücke zu stoßen, um – ebenfalls um den Preis eines Menschenlebens – fünf Menschen zu retten (Bruers und Braeckman 2014).

Verschiedentlich wurde versucht, diese offensichtlich inkonsistenten Intuitionen zu rechtfertigen. Doch es zeigt sich, dass es keine befriedigende und konsistente ethische Erklärung für die unterschiedliche Bewertung der Zulässigkeit der Handlung gibt. „Jedes Mal, wenn ein scheinbar plausibles rechtfertigendes Prinzip vorgeschlagen wurde, haben andere Philosophinnen und Philosophen Varianten zum ursprünglichen Fallpaar ersonnen, die zeigen, dass das vorgeschlagenen Prinzip unsere intuitiven Antworten nicht rechtfertigen kann.“ (Singer 2005, S. 340).

Sowohl die empirischen Befunde als auch die Geschichte der akademisch-philosophischen Auseinandersetzung mit dem Trolley-Problem deuten darauf hin, dass das ethische Problem des Gedankenexperiments sehr eng mit einem psychologischen Problem verknüpft ist. Denn während allgemein anerkannt ist, dass das Unterlassen moralisch nicht zulässiger Handlungen Vorrang vor guten Taten hat, ist die Subsumtion konkreter Handlungen unter die eine oder andere Kategorie nicht konsistent.

So betrachten viele Menschen das Verstellen der Weiche als akzeptabel. Nur ein kleiner Teil plädiert dafür, den Mann von der Brücke zu stoßen. Und kaum jemand würde akzeptieren, wenn in einem Krankenhaus, in dem fünf Menschen auf Spenderorgane warten, eine Paketbotin gekidnappt und getötet wird, weil sie eine passende Organspenderin für alle fünf ist. Doch sowohl das Verstellen der Weiche als auch das Herunterstoßen des Mannes von der Brücke ebenso wie das Töten der Paketbotin sind strukturell gleiche Taten. In allen Fällen wird aktiv ein Mensch getötet, damit wird in allen Fällen gegen das Tötungsverbot verstoßen.

Die Tatsache, dass man im einen Fall eine technische Einrichtung benutzt, in den anderen Fällen die bloßen Hände sowie ein Skalpell, führt offenbar zu einem Auseinanderfallen von subjektiv-psychologischer und objektiv-ethischer Bewertung. Sofern man jedoch den Anspruch, ethische Urteile auf rationale Argumentationen zu stützen, nicht aufgeben und kontingenten Faktoren wie Sympathie, Hautfarbe, sich mit oder ohne Fahrzeug auf den Schienen zu befinden, etc., Raum geben will, müssen die Details der rettenden Tötungshandlung unbeachtet bleiben.

### Die Pandemie und das Luftsicherheitsgesetz als Trolley-Probleme

Die gegenwärtige Situation lässt sich als direkte Analogie zum Trolley-Problem und dem damit aufgezeigten moralischen Dilemma beschreiben. In beiden Fällen ist eine unabwendbare Bedrohungssituation mit tödlichem Ausgang gegeben. Der auf eine Gruppe von Menschen zurollende Trolley entspricht dem Virus, das sich weltweit ausbreitet, gewissermaßen auf die Menschen zurollt.^12^ Den politischen Akteurinnen stellt sich die Frage nach der richtigen Handlungsweise, denn – und das scheint häufig übersehen zu werden – sowohl im Gedankenexperiment als auch in der Realität bedeutet ein Eingreifen, das Leben anderer Menschen zu opfern.

Im Trolley-Dilemma hat die zu treffende Entscheidung Einfluss auf die Zahl der Opfer, aber nicht auf die Tatsache, dass Menschen sterben. Ebenso ist es in der realen Situation. Sowohl die Infektion an sich, als auch die Verordnungen zur Verhinderung der Ausbreitung haben tödliche und andere negative Konsequenzen. Somit befinden sich die politischen Akteurinnen in einem Trolley-Dilemma. Ob sie handeln, also Ausgangssperren, Schließungen vieler Einrichtungen oder Kontaktbeschränkungen verfügen, oder nicht handeln: Menschen werden sterben.

Dieses Faktum ist in der öffentlichen Diskussion bisher nicht zur Geltung gekommen. Während die gesundheitlichen Folgen bis hin zum Tod einer Covid-19-Erkrankung den meisten Menschen deutlich vor Augen stehen, werden entsprechende Folgen der Antipandemiepolitik nicht im gleichen Maße beachtet, obschon diese ebenso real sind. Daher müssen sie in der ethischen Bewertung der bisherigen Maßnahmen ebenso berücksichtigt werden wie in der Diskussion über das weitere Handeln.

Eine theoretisch-philosophische Bewertung kann aus utilitaristischer sowie aus deontologischer Perspektive erfolgen. Utilitaristische Überlegungen können sich dabei an einem klaren Ziel orientieren: der Minimierung von Todesfällen und schweren gesundheitlichen Schäden. Wenn das den Tod von Menschen erfordert, so ist das bedauerlich, aber, sofern der Gesamtnutzen gesteigert wird, vertretbar, wenn nicht sogar erforderlich. Um fünf Menschen zu retten, darf man die Weiche verstellen, den Mann von der Brücke stoßen, oder die Paketbotin mit den passenden Organen töten.

Anders stellt sich die Sache dar, wenn man einem deontologischen Ansatz folgt. Übersetzt in das Bild des Trolley-Problems stellt sich die bisherige Politik als eine Handlung dar, die den Trolley auf dasjenige Gleis lenken soll, auf dem sich weniger Menschen befinden – so zumindest die Hoffnung. Zugleich ist klar, dass die Eindämmungspolitik neben materiellen, gesundheitlichen und anderen Kosten auch Menschenleben fordert. Mithin werden Menschen geopfert. Das ist jedoch aus deontologischer Perspektive unzulässig – je nach Theorieausprägung entweder deshalb, weil es ein moralisches Gesetz verletzt, oder, weil es die moralischen Rechte der betroffenen Menschen verletzt. Auch die Hoffnung, dass dadurch insgesamt mehr Menschen überleben, ändert nichts an der Bewertung. Folgt man also dieser Ansicht, so ist der bisherige politische Kurs ethisch nicht zulässig.

Dieser Befund ist bereits für sich genommen ernüchternd. Hinzu treten rechtliche Bedenken. Anders als philosophische Gedankenexperimente sind politische Entscheidungen keine konstruierten Situationen mit frei wählbarem Bezugsrahmen. Vielmehr werden sie innerhalb einer verbindlichen Rechtsordnung getroffen. Das bedeutet für den deutschen Kontext, dass sich politische Entscheidungen innerhalb der Werteordnung des Grundgesetzes und dessen Fundamentalnorm der Menschenwürde bewegen müssen. Damit ist nicht ausgesagt, dass Moralphilosophie und Jurisprudenz voneinander unabhängig sein sollen oder können. Sie sind allerdings auch nicht identisch.

Im konkreten Fall stellt sich die Frage, ob die Antipandemiepolitik das Gebot der Achtung der Menschenwürde verletzt. In diesem Zusammenhang ist der Verweis auf das Urteil des Bundesverfassungsgerichts zum Luftsicherheitsgesetz aufschlussreich. Das Luftsicherheitsgesetz sah in §14 Abs. 3 der 2005 gültigen Fassung die Möglichkeit vor, ein entführtes Flugzeug abzuschießen, wenn dieses wie etwa bei den Terroranschlägen vom 11. September 2001 als Waffe eingesetzt werden sollte. Diese Bestimmung bildet eine unmittelbare Parallele zu den Maßnahmen zur Eindämmung von SARS-CoV-2-Infektionen, insofern sowohl das Luftsicherheitsgesetz in der damaligen Fassung wie auch die Corona-Verordnungen die rechtliche Grundlage für Maßnahmen bilden, die jeweils voraussehbar den Tod zufällig involvierter Menschen zur Folge haben.

Das Bundesverfassungsgericht hat diese Bestimmung des Luftsicherheitsgesetzes als unvereinbar mit der Verfassung bezeichnet (1 BvR 357/05). Der Grund dafür war nicht, dass in das menschliche Leben als Freiheitsrecht eingegriffen wird, sondern dass die Umstände eines solchen Eingriffs die Menschenwürde der ansonsten unbeteiligten Flugzeuginsassen verletzen. Der Staat behandle die Flugzeuginsassen „als bloße Objekte seiner Rettungsaktion zum Schutze anderer.“ (1 BvR 357/05, Rn. 24).

Überträgt man diese Formulierung auf die gegenwärtige Situation, so bedeutet dies, dass der Staat die Menschen als bloße Objekte seiner Rettungsaktion zum Schutze anderer behandelt. Er würdigt Individuen herab zu einer vertretbaren Größe, wie es die Objektformel als wohl bekannteste Konkretisierung der Würde des Menschen formuliert (vgl. Herdegen 2019, Rn. 36). Entscheidend ist nicht länger die Person, sondern die Zahl. Der mögliche Einwand, dass die geschützten Personen und diejenigen, welche die negativen Folgen der Corona-Verordnungen erleiden müssen, dieselbe Gruppe, nämlich die gesamte Bevölkerung, darstellen, verfängt nicht. Denn dies gilt auch im gedachten Anwendungsfall von §14 Abs. 3 Luftsicherheitsgesetz a. F. Erst mit der Entführung des Flugzeugs stellt sich heraus, wer die Opfer in der Luft sind; wer die Opfer am Boden sein werden, wird sich meist zu einem noch späteren Zeitpunkt klären. Dass es sich in der Corona-Pandemie möglicherweise umgekehrt verhält, und die an Covid-19 Verstorbenen eher bekannt sind als Menschen, die auf Grund der Antipandemiemaßnahmen sterben, ist unbeachtlich.

Unbeachtlich ist auch das Problem, dass es nicht immer möglich sein wird, die Opfer der Antipandemiepolitik zu identifizieren. Das ist bei Pandemieopfern nicht anders. Nicht immer wird zu klären versucht, ob die SARS-CoV-2-Infektion bzw. die dadurch verursachte Erkrankung (mit)ursächlich für einen Todesfall war oder ob der Tod aus anderen Gründen eingetreten ist. Vor allem aber ändert eine ausbleibende individuelle Identifikation nichts an der moralischen Bewertung des zum Tode führenden Handelns.

Die unterschiedliche Wahrnehmung der jeweiligen Handlungsfolgen weist allerdings auf ein Problem hin, das Frédéric Bastiat bereits im 19. Jahrhundert sehr anschaulich beschrieben hat: die menschliche Tendenz, Offensichtliches stärker zu gewichten als Handlungsfolgen, die zwar nicht weniger bedeutend und schwerwiegend sind, aber eben nicht sogleich ins Auge springen. In seiner Parabel von der zerbrochenen Scheibe, Untertitel: was man sieht und was man nicht sieht, spießt er diesen gedanklichen Kurzschluss elegant auf (Bastiat 1874).

In der Parabel zerstört ein Junge eine Fensterscheibe. Die öffentliche Meinung sieht darin einen ökonomischen Gewinn, schließlich beschert die Reparatur der Scheibe dem Glaser Einnahmen. Allerdings kann die Auftraggeberin ihr Geld nur einmal ausgeben. Hätte sie nicht die Fensterscheibe reparieren lassen müssen, hätte sie etwas anderes kaufen können – Bücher etwa, oder Schuhe. Ob nun der Glaser oder die Schuhmacherin tätig werden, kann Außenstehenden egal sein. Problematisch ist hingegen, dass die Auftraggeberin (und wenn man die Parabel auf größere Zusammenhänge überträgt, die gesamte Gesellschaft) nun ärmer ist. Denn die Auftraggeberin musste ihr Geld für eine unnötige Reparatur ausgeben. Hätte sie sich stattdessen Schuhe kaufen können, würde sie nicht nur in ihrer Stube im Warmen sitzen können, sondern auch draußen warme Füße haben. Wegen der Zerstörung des Fensters muss sie sich nun für eines von beiden entscheiden.

In der Corona-Pandemie geht es nicht um Fensterscheiben und Schuhe, sondern um Gesundheit und Menschenleben. Umso wichtiger ist es, dass eine ethische vertretbare Betrachtung nicht auf die unmittelbare Sichtbarkeit von Schäden abstellt. Denn das würde bedeuten, ohne sachlichen Grund mit zweierlei Maß zu messen. Das ist jedoch ethisch wie rechtlich unzulässig; es gibt keinen akzeptablen Grund, die Gesundheit und das Leben derjenigen Menschen, die durch Corona-Verordnungen geschädigt werden, geringer zu achten als das Leben jener, die durch Covid-19 bedroht sind.

## Implikationen für zukünftiges Handeln

Ich habe aufgezeigt, dass und warum die gegenwärtige Situation der Corona-Pandemie als ein Dilemma aufzufassen ist, dessen moralisch zulässige Lösung nicht in einer Politik bestehen kann, die das Leben der einen auf Kosten des Lebens der anderen rettet. Dieser Befund impliziert aber weder, dass es grundsätzlich falsch oder unmöglich wäre zu handeln, noch, dass dies nicht auf politischer Ebene geschehen dürfte. Ganz im Gegenteil. Ein unreflektiertes Nichthandeln ist aus ethischer Sicht ebenfalls keine Option. Zwar mag eine Infektion mit SARS-CoV‑2 für viele Menschen harmlos oder gar ohne Symptome verlaufen, für nicht wenige ist es aber eine schwere Erkrankung mit schlimmstenfalls tödlichem Ausgang. Daher ist es aus ethischer Perspektive dringend geboten, Mittel und Wege zur Verringerung dieses Leids zu ersinnen.

Um den Spielraum für solche Maßnahmen auszuloten, ist es zunächst sinnvoll, die Unterschiede zwischen Trolley-Problem und Corona-Pandemie zu verdeutlichen. Zum einen ist das Geschehen in der Realität nicht auf einen Ort begrenzt. Das Infektionsgeschehen findet weltweit statt, wenn auch zu unterschiedlichen Zeiten und in unterschiedlicher Schwere. Eingedenk der Unterschiede sowohl zwischen allen betroffenen Ländern als auch innerhalb der Länder hinsichtlich der medizinischen Infrastruktur, Siedlungsdichte, Altersstruktur und Gesundheitszustand der Bevölkerung ist es höchst unwahrscheinlich, dass es einen idealen und für alle passenden Weg gibt. Vielmehr ist es erforderlich, das spezifische Wissen um lokale Gegebenheiten optimal zu nutzen, um bestmöglich reagieren zu können (vgl. Ostrom 1990). Daraus folgt, dass Maßnahmen sich im internationalen Vergleich ebenso wie innerhalb eines Landes unterscheiden dürfen und vielleicht sogar unterscheiden müssen, was sie bereits tun.

Zum anderen ist in der Realität, anders als im Gedankenexperiment, das Wissen um Eingriffsmöglichkeiten weder definitiv noch begrenzt. Die Suche nach einem Ausweg scheint sogar eine intuitive menschliche Reaktion zu sein. So erklärte der US-Philosoph David Schmidtz Seminarteilnehmerinnen, dass es per definitionem keine anderen Lösungsmöglichkeiten des Trolley-Dilemmas gibt. Dennoch war ihr erster Impuls die Suche nach einem Ausweg (Schmidtz 2007, S. 447). Diese hoffnungsvolle Tatsache liegt der intensiven Suche nach Impfstoffen und Gegenmitteln zu Grunde, und ebenso der Debatte über Sinn und Möglichkeit von Lockerungen der strengen Corona-Verordnungen. Logik und Erfahrung sprechen dafür, diesen Suchprozess möglichst breit und offen zu gestalten. Denn das erhöht die Wahrscheinlichkeit, Ideen im Dialog weiterzuentwickeln. Daher sind die Pluralität der vorgebrachten Ansichten ebenso wie das Engagement vieler Gruppen und Individuen in der Auseinandersetzung nicht nur Ausweis einer soliden und lebendigen freiheitlich-demokratischen Kultur, sondern geben auch Anlass zu der Hoffnung, dass immer bessere Maßnahmen zur Infektionseindämmung entwickelt werden.

Dagegen ist das Denken in der Kategorie von Öffnungsdiskussionsorgien doppelt problematisch. Einerseits kommt darin ein eindimensionales Denken zum Ausdruck, dass mit einer Lockerung oder Verschärfung der bereits implementierten Maßnahmen einen quantitativen Aspekt in den Blick nimmt, aber wenig Raum für kreative und qualitative Neuerungen lässt. Andererseits begünstigt es die Tendenz einer unreflektierten Präferenz für das Entscheiden, die auch der Rede von einer angeblichen Stunde der Exekutive zu Grunde liegt. Diese Auffassung ist jedoch nicht nur demokratietheoretisch überaus bedenklich. Sie ist auch in der Sache fatal, insofern eine außergewöhnliche Stärkung der Befugnisse der Exekutive weder für das Überleben möglichst vieler Menschen noch für die Fortexistenz einer liberalen Demokratie eine gute Strategie ist (vgl. Bjørnskov und Voigt 2018, 2020).

Auch wenn es angesichts des menschlichen Action Bias, also der Neigung zum Handeln, selbst wenn das erkennbar nutzlos oder gar schädlich ist (Patt und Zeckhauser 2000), kontraintuitiv erscheinen mag: Eine breite öffentliche Diskussion bietet die bestmögliche Gewähr dafür, dass unter der andauernden Bedingung großer Unsicherheit die in schneller Folge gewonnenen neuen Erkenntnisse bei der Lösungsfindung berücksichtigt werden können. Im Trolley-Dilemma sind alle Einflussfaktoren per definitionem gegeben. In der Realität dagegen ist keinesfalls sicher, dass eine Maßnahme den intendierten Effekt zeitigt, oder dass sich erste Hypothesen und Erkenntnisse über Virus und Erkrankung bestätigen.

Der Abgleich zwischen Gedankenexperiment und Realität zeigt, dass es durchaus Potenziale, aber auch Fallstricke für politisches Handeln in der Antwort auf die Corona-Pandemie gibt. Eine Zusammenschau der faktischen und normativen Randbedingungen beschreibt das Feld der Möglichkeiten.

Ausgangspunkt ist dabei die Analyse der Situation und des ihr innewohnenden ethischen Konflikts. Dieser besteht nach der Wahrnehmung des Ethikrats darin, die Funktionsfähigkeit des Gesundheitssystems zu sichern, und den schwerwiegenden Nebenfolgen der Maßnahmen, die diesem Ziel dienen (Deutscher Ethikrat 2020). Ein funktionierendes Gesundheitssystem kann jedoch nur solche Todesfälle vermeiden helfen, bei denen fehlende medizinische Mittel mitursächlich sind. Die Menschen, die trotz optimaler medizinischer Versorgung versterben, sind von dieser Beschreibung allenfalls mittelbar erfasst, sofern getroffene Maßnahmen sie vor einer Infektion schützen.

Demgegenüber plädiere ich dafür, nicht die Funktionsfähigkeit des Gesundheitssystems als Ziel zu betrachten, sondern die Anzahl der Todesfälle in der Folge von Covid-19 möglichst gering zu halten. Daher ist es nur von nachgeordneter Wichtigkeit, die Dynamik des Infektionsgeschehens in vermutlich nur sehr geringfügig gefährdeten Gruppen zu kontrollieren. Priorität hätte demgegenüber der Schutz besonders vulnerabler Personen.

Die konkreten Maßnahmen dürfen ganz offensichtlich auch in diesem Fall nicht gegen das Menschenwürdegebot verstoßen. Auch hier ist also eine Politik ausgeschlossen, die voraussehbar zu Todesfällen an anderer Stelle führt. Ebenso wenig darf aber die Würde der besonders zu schützenden Gruppen verletzt werden. Das impliziert, dass das Selbstbestimmungsrecht als fundamentale allgemein- und medizinethische Norm auch der am schwersten Betroffenen gewahrt werden muss (vgl. English et al. 2004, S. 71 ff). Hier kommt etwa die Schaffung von Wahlmöglichkeiten oder Angeboten von (statt Verpflichtungen zu) Infektionsschutzmaßnahmen in Betracht, sofern dies nicht zu übergroßer Fremdgefährdung führt.

Im Lebensalltag stößt dieser Anspruch auf mannigfache Barrieren, die sich beispielsweise aus der räumlichen Lebenssituation oder Betreuungspflichten für andere ergeben. Während freiwillige Telearbeit und Lebensmittellieferungen einer alleinstehenden Person vergleichsweise problemlos den Rückzug in die freiwillige Quarantäne ermöglichen, gestaltet sich dies beim Zusammenleben mit Kindern deutlich schwieriger. Noch anders stellt sich die Situation für Menschen dar, die in Einrichtungen leben. Hier müssen nicht nur organisatorische Schwierigkeiten gelöst werden, sondern unter Umständen unterschiedliche Prioritäten, etwa fortdauernder sozialer Kontakt versus maximale Selbstisolation, miteinander in Einklang gebracht werden.

Während die Bedeutung einer peniblen Hygiene außer Frage steht und der Wunsch nach Schutz vor einer Infektion fraglos respektiert wird, stellt die Achtung der Selbstbestimmung anderer, zumal wenn diese besonders vulnerabel erscheinen, eine Herausforderung an die Toleranz dar. Das normative Postulat, „[j]eder Mensch hat das Recht, Entscheidungen, die seine Gesundheit betreffen, selbstbestimmt zu treffen – selbst wenn diese Entscheidungen Dritten unvernünftig erscheinen“ (DGPPN 2014, S. 3), gilt unbedingt. Es ist nicht durch die Limitationen Dritter einzuschränken, sondern allenfalls durch Einschränkungen der Selbstbestimmungsfähigkeit der betroffenen Person (DGPPN 2014). Daraus folgt, dass wer in dem Wissen um möglicherweise nicht ausreichende medizinische Kapazitäten das Risiko einer SARS-CoV-2-Infektion eingehen möchte, dazu jedes Recht hat. Schließlich sind Rauchen, Motorradfahren und einseitige sitzende Tätigkeiten wie das Schreiben von Büchern auch nicht verboten, obschon mit einem gewissen Potenzial ausgestattet, viele medizinische Ressourcen zu absorbieren und womöglich dennoch das Leben zu verkürzen. Selbst bei Angehörigen von Risikogruppen ist zu tolerieren, dass diese ihr Leben nach eigenen Vorstellungen leben.

Diese Toleranz wird bedauerlicherweise oft missverstanden, wie Robert Nozick in seiner Analyse der normativen Grenzen der Staatstätigkeit feststellt. „Obwohl nur die mit Zwang verbundenen, nicht die freiwilligen Wege zu diesen Zielen ausgeschlossen werden, dürften viele Menschen unsere Ergebnisse auf der Stelle ablehnen, weil sie eine so offensichtliche Gleichgültigkeit gegenüber den Bedürfnissen und dem Leiden anderer einfach nicht akzeptieren wollen. Ich kenne diese Reaktion; sie stellte sich auch bei mir ein, als ich solche Auffassungen zu betrachten begann.“ (Nozick 2006, S. 13) Tatsächlich ist es aber nicht Gleichgültigkeit, sondern im Gegenteil die Achtung vor dem Leben anderer Menschen, die der Anwendung von Zwang eine Grenze setzt.

## Schluss

Öffentliche Orte, die sonst belebt sind, sind leergefegt. Messe- oder Veranstaltungshallen werden innerhalb kürzester Zeit zu improvisierten Krankenhäusern umgebaut. Särge werden mit Militärlastwagen abtransportiert, und Menschen werden in eilig ausgehobenen Behelfsgräbern bestattet. Täglich werden Zahlen neuer SARS-CoV-2-Infektionen und Todesfälle vermeldet. Solche verstörenden Informationen und Bilder begleiten uns seit Wochen.

Es steht außer Frage, dass es politisch ebenso wie ethisch geboten ist, dieses Leid so gut als möglich zu lindern. In Ermangelung von Impfstoffen und Therapeutika bleibt als Lösung jedoch nur, Infektionen zu vermeiden. Zugleich müssen die Maßnahmen zur Infektionsvermeidung ethisch vertretbar sein. Das bedeutet zuallererst, dass die Maßnahmen die Menschenwürde nicht verletzen dürfen, etwa dadurch, dass Menschen zu Objekten staatlicher Fürsorge degradiert werden oder dass die Maßnahmen selbst Todesfälle verursachen.

In diesem Text habe ich argumentiert, dass die im März und April 2020 in Deutschland implementierte Antipandemiepolitik dieser Anforderung nicht gerecht wird. Insofern trifft es nicht den Kern des Problems, die Maßnahmen unter Verhältnismäßigkeitsgesichtspunkten zu diskutieren. Der weitgehende Lockdown mag Infektionen verhindern, er hat aber zugleich selbst erhebliche negative gesundheitliche Auswirkungen. Diese ergeben sich z. T. direkt aus den verfügten Einschränkungen, wie ein verbreiteter Bewegungsmangel, der als führende Ursache für einige Krebsarten, Schlaganfälle oder Bluthochdruck (ironischerweise ein Risikofaktor für einen schweren Covid-19-Verlauf) und daraus resultierende Todesfälle gilt. Andere Auswirkungen kommen indirekt zum Tragen, etwa in der Form von Rezession und erhöhter Arbeitslosigkeit, was mit steigenden Suizidraten respektive kürzerer Lebenserwartung assoziiert ist.

Konflikte wie der vorliegende werden vielfach anhand des Trolley-Dilemmas oder verwandter Gedankenexperimente diskutiert. Im Kern geht es immer um die Frage, ob man Menschen opfern darf, um andere Menschen zu retten. Sofern man sich nicht auf ein utilitaristisches Aufrechnen von Menschenleben einlassen möchte, ist die Antwort eindeutig: Das Retten von Menschenleben taugt nicht zur Rechtfertigung von Maßnahmen, die vorhersehbar den Tod von Menschen zur Folge haben.

Um ein weiteres Bild aus der Trolley-Familie zu bemühen: Wenn man an einer Küste entlangfährt, und vor der herannahenden Flut entweder eine einzelne Person retten kann oder aber fünf Personen, ist es statthaft, sich für die Rettung der fünf zu entscheiden. Muss man aber dafür die eine Person überfahren, ist die Rettung der fünf nicht zulässig. Denn man würde dadurch die eine Person herabwürdigen zu einem Hindernis, das der Rettung anderer im Wege steht. Damit aber würde man ihre Menschenwürde verletzen (Foot zitiert nach Hacker-Wright 2019).

Auch dann, wenn man nicht auf die Menschenwürde abstellt, sondern von einem absoluten und unantastbaren Wert des menschlichen Lebens ausgeht, kommt man zu keinem anderen Ergebnis. Denn das daraus abgeleitete Gebot, Leben zu schützen, würde sich selbst ad absurdum führen, wenn man deshalb Menschen töten oder tödlichen Risiken aussetzen würde. Auch kann es weder normativ noch praktisch bedeuten, dass jedes Lebensrisiko auszuschließen ist. Vielmehr geht es darum, besonders gravierende Eingriffe in das Leben, wie z. B. Straftaten oder aber das Opfern von Menschen zur Rettung anderer, zu verhindern.

Dass die deutsche Politik hier nicht konsequentialistisch, also mit einer möglicherweise größeren Zahl Geretteter, argumentieren darf, hat das Bundesverfassungsgericht in seinem Urteil zum Luftsicherheitsgesetz klargestellt. Ursprünglich sah das Gesetz vor, dass ein entführtes Flugzeug, das als Waffe gebraucht werden sollte, als ultima ratio zur Verhinderung vieler weiterer Todesopfer abgeschossen werden durfte. Obschon also in einer solchen Situation das Opfern einer vergleichsweise geringen Zahl von Menschen, denen überdies nach menschlichem Ermessen ohnehin nur noch eine sehr kurze Lebensspanne bleibt, die Rettung einer Vielzahl anderer Menschen ermöglicht, ist ein solches Vorgehen unethisch und verfassungswidrig.

Unabhängig von ethischen Erwägungen bringen solche Dilemmata in der Realität eine große psychische Belastung mit sich: Ohnmacht angesichts der existentiellen Bedrohung menschlichen Lebens. Insofern ist es nicht überraschend, dass Menschen sowohl dazu neigen, wider besseres Wissen zu handeln, und dieses Handeln darüber hinaus zu rechtfertigen suchen. Glücklicherweise gibt es in der gegenwärtigen Situation der Corona-Pandemie nicht nur zwei Optionen. Es wurden bereits unterschiedlichste Maßnahmen in Kraft gesetzt, und weitere können entwickelt werden. Insofern ist es Chance und Verpflichtung, nach solchen Maßnahmen zu suchen, die die Zahl der an Covid-19 erkrankenden und versterbenden Menschen minimieren, ohne dabei die Gesundheit, das Leben und die Menschenwürde der den Maßnahmen Unterworfenen zu verletzen.

## Footnotes

1 Stand: 21. April 2020, https://coronavirus.jhu.edu/data/mortality. Letalität ist dabei als case fatality ratio, also Anteil der Verstorbenen an allen Fällen, definiert.

2 Ein internes Papier des Bundesinnenministeriums stellt als Worst-Case-Szenario eine Millionen Tote in Aussicht, wobei eine Letalität von 1,2–2 % zu Grunde gelegt wird. Dieses Papier ist nicht veröffentlicht worden. Es liegt nach eigenen Angaben diversen Medien vor, das Informationsfreiheitsprojekt „Frag’ den Staat“ gibt an, dass ein von ihm veröffentlichtes Dokument authentisch ist: https://fragdenstaat.de/dokumente/4123-wie-wir-covid-19-unter-kontrolle-bekommen/.

3 Stand: 21. April 2020, https://coronavirus.jhu.edu/data/mortality.

4 Während in Fauci et al. (2020) die Letalität von Covid-19 als näher an derjenigen einer saisonalen oder pandemischen Influenza (0,1 %) als an derjenigen von SARS (9–10 %) oder gar MERS (36 %) beschrieben wird, nutzt Fauci in öffentlichen Statements oft die Formel, Covid-19 sei zehn Mal tödlicher als eine saisonale Grippe (Flatley 2020).

5 Zum Vergleich: in den vergangenen Jahren sind in Deutschland jährlich zwischen 900.000 und 1.000.000 Menschen gestorben (Statistisches Bundesamt 2017, 2020).

6 Grundlage der hier entfalteten Interpretation sind Diskussionsbeiträge und Begründungen, die oftmals sehr kurz und thesenartig sind. Insofern ist es vorstellbar, dass Einzelne sehr wohl von einer absoluten Pflicht zur Lebensrettung ausgehen. Auch dann stellt sich jedoch das Problem, dass die Lebensrettung verwehrt ist, wenn dafür andere Menschen getötet werden müssen – wenn eine solche absolute Pflicht existiert, dann gilt sie gegenüber jedermann.

7 Kurz nach Fertigstellung des Manuskripts sind Beiträge erschienen, in denen das hier erörterte ethische Problem zumindest kurz angerissen wird (Höffe 2020; Hesse und Merkel 2020). Sie haben nach meiner Wahrnehmung die Debatte in Politik und Öffentlichkeit jedoch nicht maßgeblich beeinflusst. Vielmehr wird im Oktober 2020 ein erneuter Lockdown diskutiert.

8 Selbstredend kann die Situation auch so gedeutet werden, dass die negativen Folgen gezielt ignoriert werden, und damit eine bewusst unethische Politik verfolgt wird. Diese Option erörtere ich im Text jedoch nicht, sondern gehe von der Gutwilligkeit aller Akteurinnen und Akteure aus.

9 In der Tat ist diese kleine Veränderung entscheidend für die Beurteilung der Handlung. Während eine Straßenbahnfahrerin in solch einer Situation in jedem Fall töten wird, und es daher zulässig ist, dass sie sich dafür entscheidet, nur eine Person zu überfahren, muss eine Passantin zwischen einem zulässigen Sterbenlassen und einem unzulässigen Töten wählen. Die Intuition, dass es für die Passantin zulässig sei, die Weiche umzustellen, da in der Folge weniger Menschen sterben, lässt sich nicht konsistent begründen, sofern man nicht grundsätzlich bereit ist, die moralische Pflicht, andere Menschen nicht zu töten, aufzugeben (Thomson 2008).

10 Die Engführung resultiert daher, dass das Trolley-Dilemma um genau dieser Kontrastierung willen konstruiert wurde. Andere ethische Theorien wie z. B. ethischer Egoismus sind daher nicht einschlägig oder bedürfen, wie die Tugendethik, weiterer Konkretisierungen, um zu einem Urteil zu kommen. Eine Ausnahme wäre allenfalls eine hybride Theorie, die zwar konsequentialistisch argumentiert, allerdings die Nichtverletzung moralischer Rechte o. ä. zumindest als Bestandteil des zu maximierenden Guten begreift.

11 Deontologie wird hier im weiten Sinne verwandt und bezieht sich auf eine Theoriefamilie, nicht etwa ausschließlich auf Kant. Ethische Pflichten können somit auch aus moralischen Rechten von Personen resultieren (vgl. die programmatische Formulierung Nozicks: „Die Menschen haben Rechte, und einiges darf ihnen kein Mensch und keine Gruppe antun“ 2006, S. 13). Unterschiedliche Positionen finden sich auch bezüglich der Frage, ob und wenn ja, inwieweit vorhersehbare Handlungsfolgen im Falle von Normkonflikten als Hilfskriterien herangezogen werden können. Daher unterscheiden sich die deontologischen Begründungen sowie die Erstreckung des Tötungsverbots; in Fällen wie der Tötung auf Verlangen oder Selbstmord gehen die Ansichten auseinander. Für Fälle, in denen keine Einwilligung vorliegt und vielmehr das Gegenteil vermutet werden muss, ist es jedenfalls unstrittig.

12 Allerdings besteht ein Unterschied in der Letalität. Während der Trolley in der hypothetischen Situation zu 100 % tödlich ist, ist in der Realität weder klar, wie viele der Infizierten erkranken, noch, wie viele der Erkrankten versterben.

## References

[CR1] Altenbockum, Jasper von. 2020. „Ausgangsbeschränkungen: Es geht nur mit dem Hammer“. Frankfurter Allgemeine Zeitung. https://www.faz.net/1.6688646. Zugegriffen: 2. Mai 2020.

[CR2] Bastiat, Frédéric. 1874. The broken window. In *Essays on political economy. By the late M. Frédéric Bastiat*, 50–54. : G. P. Putnams & Sons.

[CR3] Birnbaum, Robert, und Georg Ismar. 2020. „Schäuble will dem Schutz des Lebens nicht alles unterordnen“. Der Tagesspiegel. https://www.tagesspiegel.de/politik/bundestagspraesident-zur-corona-krise-schaeuble-will-dem-schutz-des-lebens-nicht-alles-unterordnen/25770466.html. Zugegriffen: 27. Apr. 2020.

[CR4] Bjørnskov, Christian, und Stefan Voigt. 2018. „More power to government = more people killed?—On some unexpected effects of constitutional emergency provisions during natural disasters“. SSRN scholarly paper. https://papers.ssrn.com/abstract=3189749. Zugegriffen: 22. Apr. 2020.

[CR5] Bjørnskov, Christian, und Stefan Voigt. 2020. „The state of emergency virus“. streeck. https://verfassungsblog.de/the-state-of-emergency-virus/. Zugegriffen: 21. Apr. 2020.

[CR6] Blom, Annelies G., et al. 2020. Die Mannheimer Corona-Studie: Das Leben in Deutschland im Ausnahmezustand. Bericht zur Lage vom 20. März bis 21. April 2020. Mannheim. https://www.uni-mannheim.de/media/Einrichtungen/gip/Corona_Studie/22-04-2020_Mannheimer_Corona-Studie_-_Bericht_zur_Lage_in_den_Tagen_20_Mrz-21_Apr_2020.pdf. Zugegriffen: 22. Apr. 2020.

[CR7] Bruers S, Braeckman J (2014). A review and systematization of the trolley problem. Philosophia.

[CR8] Bundesagentur für Arbeit. 2020. Monatsbericht zum Arbeits- und Ausbildungsmarkt April 2020. https://www.arbeitsagentur.de/datei/ba146454.pdf. Zugegriffen: 6. Mai 2020.

[CR9] Chang S-S, Stuckler D, Yip P, Gunnell D (2013). Impact of 2008 global economic crisis on suicide: time trend study in 54 countries. British Medical Journal.

[CR10] Cheng, Cindy, et al. 2020. CoronaNet: a dyadic dataset of government responses to the COVID-19 pandemic. https://osf.io/dkvxy. Zugegriffen: 26. Apr. 2020. SocArXiv.

[CR12] Deutsche Gesellschaft für Psychiatrie und Psychotherapie,Psychosomatik und Nervenheilkunde e. V. (DGPPN) (2014). Achtung der Selbstbestimmung und Anwendung von Zwang bei der Behandlung psychisch erkrankter Menschen: Eine ethische Stellungnahme der DGPPN. Der Nervenarzt.

[CR13] Deutscher Ethikrat (2020). Solidarität und Verantwortung in der Corona-Krise. Ad-hoc-Empfehlung.

[CR55] Duke AA, Bègue L (2015). The Drunk Utilitarian: Blood Alcohol Concentration Predicts Utilitarian Responses in Moral Dilemmas. Cognition.

[CR14] Edmonds D (2013). Would you kill the fat man?—The trolley problem and what your answer tells us about right and wrong.

[CR15] Encke A, Haas S, Kopp I (2015). S3-Leitlinie Prophylaxe der venösen Thromboembolie (VTE).

[CR16] English V, Romano-Critchley G, Sheather J, Summerville A (2004). Medical ethics today. The BMA’s handbook of ethics and law.

[CR17] Fauci AS, Lane HC, Redfield RR (2020). Covid-19—Navigating the uncharted. New England Journal of Medicine.

[CR18] Flatley, Daniel. 2020. Coronavirus is 10 times deadlier than seasonal flu, Fauci says. Bloomberg.com. https://www.bloomberg.com/news/articles/2020-03-11/fauci-warns-coronavirus-far-more-lethal-than-seasonal-flu. Zugegriffen: 30. Apr. 2020.

[CR19] Fleischhauer, Jan. 2020. Rhetorik der Angst: Wie die Politik versucht, die Bürger in der Corona-Starre zu halten. FOCUS Online. https://www.focus.de/politik/deutschland/schwarzer-kanal/die-focus-kolumne-von-jan-fleischhauer-rhetorik-der-angst-wie-die-politik-die-buerger-in-der-corona-starre-zu-halten-versucht_id_11896042.html. Zugegriffen: 22. Apr. 2020.

[CR20] Foot P (1978). The problem of abortion and the doctrine of double effect. Virtues and vices and other essays in moral philosophy.

[CR21] Frühstücksfernsehen. 2020. Oberbürgermeister Boris Palmer spricht über die deutsche Wirtschaft. https://www.sat1.de/tv/fruehstuecksfernsehen/video/202082-oberbuergermeister-boris-palmer-spricht-ueber-die-deutsche-wirtschaft-clip. Zugegriffen: 4. Mai 2020.

[CR22] Gerszon Mahler, Daniel, Christopher Lakner, R. Andres Castaneda Aguilar, und Haoyu Wu. 2020. The impact of COVID-19 (coronavirus) on global poverty: why sub-saharan Africa might be the region hardest hit. https://blogs.worldbank.org/opendata/impact-covid-19-coronavirus-global-poverty-why-sub-saharan-africa-might-be-region-hardest. Zugegriffen: 6. Mai 2020.

[CR23] Guthold R, Stevens GA, Riley LM, Bull FC (2018). Worldwide trends in insufficient physical activity from 2001 to 2016: a pooled analysis of 358 population-based surveys with 1·9 million participants. The Lancet Global Health.

[CR56] Hacker-Wright, John. 2019. Philippa Foot. In *The Stanford Encyclopedia of Philosophy*, Hrsg. Edward N. Zalta, Fall 2019. Metaphysics Research Lab, Stanford University. https://plato.stanford.edu/archives/fall2019/entries/philippa-foot/. Zugegriffen: 3. Mai 2020.

[CR24] Hawton K, Haw C (2013). Economic recession and suicide. BMJ : British Medical Journal.

[CR25] Herdegen M, Herzog R, Scholz R, Herdegen M, Klein HH (2019). Art. 1. Grundgesetz. Kommentar.

[CR26] Hesse, Michael, und Reinhard Merkel. 2020. Auswirkungen der Krise: ‚Wir werden harte Verteilungskämpfe kriegen‘. Frankfurter Rundschau, 11. Mai 2020, Abschn. Kultur. https://www.fr.de/kultur/gesellschaft/rechtsphilosoph-reinhard-merkel-folgen-corona-krise-wir-werden-harte-verteilungskaempfe-kriegen-13757222.html. Zugegriffen: 11. Okt. 2020.

[CR27] Höffe O (2020). „Ist das Recht auf Leben ein Trumpf, der alles sticht? Auch dann, wenn er die Würde des Menschen tangiert? Ein paar Fragen zur Corona-Krise.

[CR28] Hurrelmann, Klaus, und Dieter Dohmen. 2020. Corona-Krise verstärkt Bildungsungleichheit. Das Deutsche Schulportal. https://deutsches-schulportal.de/stimmen/das-deutsche-schulbarometer-hurrelmann-dohmen-corona-krise-verstaerkt-bildungsungleichheit/. Zugegriffen: 12. Mai 2020.

[CR29] Hüsgen, Simon. 2020. Nach umstrittenem TV-Auftritt: Palmer bittet um Entschuldigung. Frankfurter Allgemeine Zeitung. https://www.faz.net/1.6746537. Zugegriffen: 4. Mai 2020.

[CR30] Klöpper, Anna. 2020. Freiheitsrechte und Covid-19: Die Diskussion beginnt. Die Tageszeitung: taz. https://taz.de/!5673056/. Zugegriffen: 25. Apr. 2020.

[CR31] Lüpke, Geseko von. 2020. Historiker über Demokratie und Corona: ‚Rendezvous mit dem Polizeistaat‘. Die Tageszeitung: taz. https://taz.de/. Zugegriffen: 22. Apr. 2020.

[CR32] Merkel, Angela. 2020. Fernsehansprache von Bundeskanzlerin Angela Merkel. https://www.bundeskanzlerin.de/resource/blob/260162/1732182/d4af29ba76f62f61f1320c32d39a7383/fernsehansprache-von-bundeskanzlerin-angela-merkel-data.pdf. Zugegriffen: 30. Apr. 2020.

[CR33] Neurologen und Psychiater im Netz. 2020. Anspannung und Aggression: Die Coronakrise belastet die Psyche. https://www.neurologen-und-psychiater-im-netz.org/psychiatrie-psychosomatik-psychotherapie/news-archiv/meldungen/article/anspannung-und-aggression-die-coronakrise-belastet-die-psyche/. Zugegriffen: 12. Mai 2020.

[CR34] Nozick R (2006). Anarchie, Staat, Utopia.

[CR35] Ostrom E (1990). Governing the commons: the evolution of institutions for collective action.

[CR36] Patt A, Zeckhauser R (2000). Action bias and environmental decisions. Journal of Risk and Uncertainty.

[CR37] Redaktionsnetzwerk Deutschland. 2020. Kinder und Corona: Sind sie die großen Verlierer der Krise? https://www.rnd.de/familie/kinder-und-corona-sind-sie-die-grossen-verlierer-der-krise-FT2TVFGDCGR3N6GM4GIY4DUJEQ.html. Zugegriffen: 6. Mai 2020.

[CR38] Sallis JF (2016). Progress in physical activity over the olympic quadrennium. The Lancet.

[CR39] Schmid Noerr, Gunzelin. 2020. Die Würde des Menschen ist unverrechenbar. https://www.fr.de/kultur/gesellschaft/corona-krise-wuerde-menschen-unverrechenbar-13636694.html. Zugegriffen: 29. Apr. 2020.

[CR40] Schmidtz D (2007). When justice matters. Ethics.

[CR41] Scholz R, Schulz A (2007). Zum Zusammenhang von Arbeitslosigkeit, Krankheit und Lebenserwartung. DRV-Schriften.

[CR42] Singer P (2005). Ethics and intuitions. The Journal of Ethics.

[CR43] Stalinski, Sandra. 2020. Corona und Psyche: Größere Belastung – weniger Therapien. https://www.tagesschau.de/corona-psyche-101.html. Zugegriffen: 12. Mai 2020.

[CR44] Statistisches Bundesamt (2017). Gesundheit. Todesursachen in Deutschland 2015.

[CR45] Statistisches Bundesamt. 2020. Todesursachen. https://www.destatis.de/DE/Themen/Gesellschaft-Umwelt/Gesundheit/Todesursachen/_inhalt.html. Zugegriffen: 30. Apr. 2020.

[CR46] Streeck, Hendrik, Gunther Hartmann, Martin Exner, und Matthias Schmid. 2020. Vorläufiges Ergebnis und Schlussfolgerungen der COVID-19 Case-Cluster-Study (Gemeinde Gangelt). https://www.land.nrw/sites/default/files/asset/document/zwischenergebnis_covid19_case_study_gangelt_0.pdf. Zugegriffen: 2. Mai 2020.

[CR47] swr.online. 2020. SWR Aktuell. https://www.swr.de/swraktuell/baden-wuerttemberg/landesregierung-bw-corona-krise-100.html. Zugegriffen: 4. Mai 2020.

[CR48] Tännsjö T (2013). Understanding ethics.

[CR49] Thomson JJ (1976). Killing, letting die, and the trolley problem. The Monist.

[CR50] Thomson JJ (1985). The trolley problem. Yale Law Journal.

[CR51] Thomson JJ (2008). Turning the trolley. Philosophy & Public Affairs.

[CR52] Waldmann MR, Dieterich JH (2007). Throwing a bomb on a person versus throwing a person on a bomb: intervention myopia in moral intuitions. Psychological Science.

[CR53] Warburton D (2010). A systematic review of the evidence for Canada’s physical activity guidelines for adults. International Journal of Behavioral Nutrition and Physical Activity.

[CR54] WHO. 2010. Global recommendations on physical activity for health. https://www.who.int/dietphysicalactivity/global-PA-recs-2010.pdf. Zugegriffen: 5. Mai 2020. 26180873

[CR11] YouGov. 2020. COVID-19 Tracker: Ergebnisse Deutschland Mitte April. https://yougov.de/news/2020/04/15/covid-19-tracker-ergebnisse-deutschland-mitte-apri/. Zugegriffen: 22. Apr. 2020. YouGov: What the world thinks.

